# Unconventional Ingredients in Gummy Reformulation: A Review of Nutritional, Functional, and Technological Implications

**DOI:** 10.3390/foods15152615

**Published:** 2026-07-26

**Authors:** Isabel Gonzales-Quispe, Rebeca Salvador-Reyes, Marcio Schmiele, Luz Maria Paucar-Menacho

**Affiliations:** 1 Programa de Doctorado en Ingeniería Agroindustrial, Mención Transformación Avanzada de Granos y Tubérculos Andinos, Universidad Nacional del Santa, Nuevo Chimbote 02712, Peru; 2025818020@uns.edu.pe (I.G.-Q.); rsalvador@uns.edu.pe (R.S.-R.); 2 Laboratório Integrado de Cereais e Lipídeos (LICEL), Instituto de Ciência e Tecnologia (ICT), Universidade Federal dos Vales do Jequitinhonha e Mucuri (UFVJM), Diamantina 39100-000, Brazil; marcio.sc@ict.ufvjm.edu.br; 3 Departamento de Ingeniería Agroindustrial y Agrónoma, Facultad de Ingeniería, Universidad Nacional del Santa, Nuevo Chimbote 02712, Peru

**Keywords:** gummy confections, food reformulation, non-conventional ingredients, functional foods, bioactive compounds, coproducts, gelling agents

## Abstract

Conventional gummies are characterized by high sugar, sweetener, and additive content, which has increased the demand for healthier alternatives. In this context, the incorporation of unconventional ingredients has emerged as a promising strategy to improve nutritional profile and functional value. This review aimed to collect and analyze information on the nutritional, technological, and sensory properties of unconventional ingredients, such as fruits, vegetables, microalgae, natural sweeteners, coproducts, and other functional compounds, in gummy formulations. A literature search was conducted, from which 20 relevant studies were selected and analyzed. The results showed that fruit incorporation increased the content of fiber, vitamin, and antioxidant compounds, while vegetables improved the mineral composition and the presence of bioactive compounds. Similarly, agro-industrial coproducts were identified as valuable sources of functional compounds with potential for sustainable valorization. In addition, the alternative gelling agents exhibited favorable effects on the texture, gel-forming capacity, stability, and shelf life of the samples. Overall, these ingredients demonstrated nutritional, technological, sensory, and sustainability advantages. However, challenges related to industrial scalability and sensory evaluation remain.

## 1. Introduction

Confectionery products have a long-standing tradition of consumption across different regions of the world, among which gummy candies stand out due to their high sensory acceptance, convenience, and growing popularity [1,2]. In recent years, this segment has shown sustained expansion in the global market. According to Euromonitor [3], the confectionery market has followed an upward trend, surpassing 680 billion USD in 2024.

Consumer preferences have evolved from purely recreational products, traditionally characterized by high levels of sugars and artificial colorants, to alternatives that also provide nutritional value and functional benefits. Gummy candies have emerged as an attractive matrix for the incorporation of innovative ingredients aimed at promoting health and well-being among consumers of different age groups [4].

Unconventional ingredients can be defined as those not commonly used in conventional gummy formulations but whose incorporation may enhance the nutritional and functional profile of the product. These ingredients can provide dietary fiber, vitamins, minerals, natural pigments, and antioxidant-active bioactive compounds [5].

**Figure 1:**
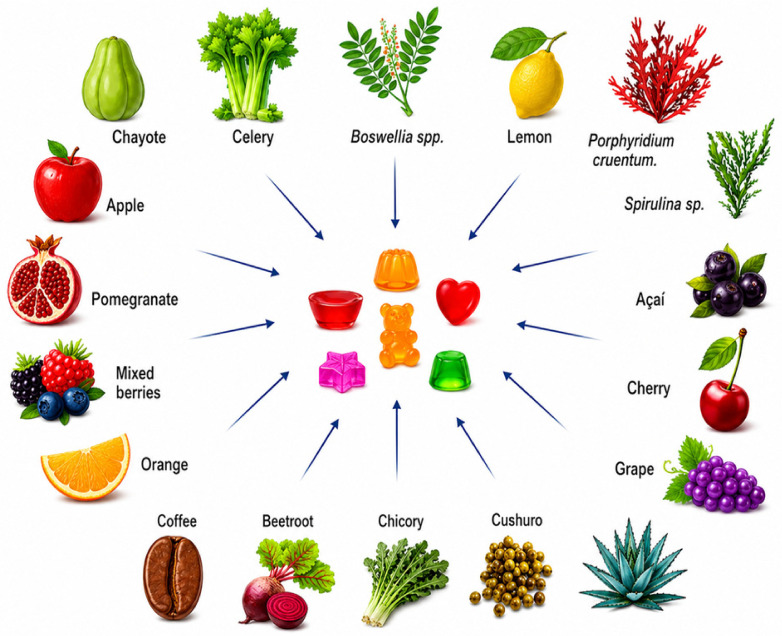
Unconventional ingredients used in gummy reformulation.

Among them are fruit and vegetable extracts, algae, plant-based sources with gelling capacity, natural sweeteners, and raw materials derived from sustainable sources (Figure 1). Although these ingredients have been widely used in other food sectors, such as beverages and bakery products, their incorporation into gummy formulations remains limited. In addition to their nutritional contribution, these ingredients can significantly influence the technological and sensory properties of gummy candies, including texture, color, stability, aroma, and overall acceptability [2].

Therefore, the development of reformulated gummy candies using unconventional ingredients requires not only the appropriate selection of raw materials but also the optimization of processing conditions to achieve a balance between technological quality, acceptability, aroma, and flavor [6].

Several studies have explored the formulation of gummies based on orange juice and berry puree [7], their enrichment with nutritionally valuable raw materials such as chayote [8], and the development of sucrose-free products without artificial colorants by incorporating ingredients such as *Spirulina* sp. and *açaí* [9].

Despite these advances, comprehensive reviews that systematically compile information on the use of unconventional ingredients in gummy reformulation are still lacking. In this regard, further research in this field is needed, considering the growing demand for products aligned with current market trends [9], which prioritize the incorporation of bioactive compounds (such as antioxidants, polyphenols, and carotenoids) and essential nutrients, including carbohydrates, proteins, lipids, dietary fiber, vitamins, and minerals [10].

Accordingly, the present study aims to compile and analyze studies on the use of unconventional ingredients in gummy reformulation and to evaluate their impact on the functional and nutritional properties of the final product.

## 2. Literature Search

This study was conducted as a narrative literature review to synthesize the current scientific evidence on the use of unconventional ingredients in gummy confectionery reformulation and their nutritional, functional, and technological implications.

The literature search was performed between September 2025 and February 2026, primarily using the Scopus database because of its extensive coverage of peer-reviewed journals in the food science and technology field. Additional manual searches were conducted by screening the reference lists of relevant articles to identify studies that had not been retrieved during the initial database search.

The search strategy used Boolean operators to combine terms related to gummy confectionery and unconventional ingredients. The main search string included the following combinations: (“gummy” OR “gummies” OR “gummy candy” OR “gummy confectionery”) AND (“fruit” OR “vegetable” OR “plant” OR “extract” OR “juice” OR “by-product” OR “coproduct” OR “seaweed” OR “macroalgae” OR “microalgae”). Additional searches were performed using more specific combinations, including “gummies AND fruits”, “gummies AND by-products”, “gummies AND vegetables”, and “gummies AND seaweed OR microalgae”, in order to maximize the retrieval of relevant studies.

Studies were considered eligible if they met the following criteria: (i) original research articles published in peer-reviewed journals; (ii) studies reporting the incorporation of one or more unconventional ingredients into gummy formulations; (iii) studies evaluating at least one nutritional, functional, physicochemical, technological, or sensory property of the developed gummies; and (iv) articles written in English. We excluded review articles, conference abstracts, book chapters, patents, duplicate records, and studies unrelated to gummy confectionery or not involving ingredient reformulation. No publication date restrictions were applied, and 20 original studies that met all the inclusion criteria were ultimately selected for qualitative synthesis.

Data regarding the unconventional ingredient incorporated, study objectives, number and composition of the formulations evaluated, and main nutritional, functional, physicochemical, technological, and sensory outcomes were extracted from each selected study. Table 1 summarizes these data, which served as the basis for organizing and critically analyzing the current evidence on unconventional ingredients used in gummy confectionery reformulation.

## 3. Fruits

Fruits are characterized by their high content of vitamins, minerals, and bioactive compounds, such as antioxidants and anti-inflammatory compounds, which have protective health effects [28]. They also represent an important source of energy and nutrients [29] and may provide up to 24% of free sugars, depending on their nature and degree of ripeness [30].

Fruits and their derivatives (e.g., juices or purées) have demonstrated high potential as coloring and flavoring agents for functional food formulation [31,32]. Their incorporation into matrices, such as gummy candies, not only contributes to the intake of bioactive compounds but also improves sensory characteristics, including texture, flavor, color, and antioxidant activity [33]. These functional effects are closely related to the structural composition of fruits, particularly their pectin, soluble sugars, organic acids, dietary fiber, and phenolic compounds content. These constituents interact with the hydrocolloid matrix of gummies, influencing gel formation, water retention, texture, color stability, and bioactive compound retention during processing [34].

Consequently, the development of gummies enriched with anthocyanins, phenolic compounds, and other bioactive metabolites has increased interest in the use of fruits as key ingredients in gummy reformulation [28].

A representative example is the study by Teixeira-Lemos et al. [7], who evaluated two gummy formulations: one prepared with orange juice slightly sweetened with honey (ORH) and another based on berry purée without the addition of sweeteners (BEM).

The results showed that both formulations exhibited low caloric content (74 kcal/100 g for ORH and 40 kcal/100 g for BEM), as well as a significant contribution of macronutrients, mainly proteins and carbohydrates. The BEM formulation stood out for its high antioxidant capacity (up to 84 mg TE/L). Both formulations showed acceptable levels of flavor, color, and texture, suggesting their potential as healthier alternatives to conventional gummies [7].

However, the authors highlight the need for further studies on stability, shelf life, process scalability, and packaging conditions to assess their feasibility for industrial applications.

Similarly, pomegranate and apple are widely used in gummy reformulation. Cano-Lamadrid et al. [11] developed 12 formulations based on pomegranate juice, apple purée, and different sweetening systems (sucrose or honey). The results showed that the incorporation of pomegranate juice led to gummies with an intense red color and enhanced brightness, while the addition of apple purée promoted the development of a softer and firmer texture, attributed to its high pectin content. This effect can be explained by the ability of pectin to form a 3D gel network through molecular interactions with sugars and acids, thereby increasing water retention and strengthening the gummy structure [35].

Furthermore, the formulation composed of 20% gelatin, pure pomegranate juice, 1% citric acid, and sucrose exhibited the highest antioxidant capacity and total polyphenol content compared with the control formulations. Sensory analysis indicated that the samples obtained scores above 5.0 on a nine-point hedonic scale, suggesting good consumer acceptability [11].

Blueberries, recognized for their high concentrations of anthocyanins, flavonoids, phenolic acids, and other phytochemicals, have been associated with beneficial effects on the gut microbiota [36], positioning them as a promising alternative to gummy reformulation. Al-Jaloudi et al. [12] developed five formulations based on blueberry concentrate and honey to obtain products with functional properties.

The results showed that the incorporation of blueberry concentrate increased the content of phenolic compounds and flavonoids in the gummies, reaching values of up to 148 mg GAE·g^−1^ and 0.51 mg RE·g^−1^, respectively. In addition, the highest antioxidant activity was observed in the formulation with a honey-to-blueberry concentrate ratio of 1:2 (IC_50_ > 100 mg/mL) [12].

However, sensory analysis indicated that the formulation with a 1:1 ratio was the most preferred by consumers, suggesting the need to balance functional benefits with the sensory acceptability of the product.

Black sapote (*Diospyros digyna* Jacq.) is a source of pigments with nutraceutical potential [37]. Jiménez-González et al. [13] obtained these compounds through fermentation using lactic acid bacteria or yeasts, followed by their extraction and application as pigments in gummy bear formulations. Four formulations with increasing pigment concentrations (1–5%) were evaluated.

The results showed that increasing pigment concentration led to significant changes in product color, ranging from light brown to nearly black tones (1%) to nearly black (5%), accompanied by a decrease in lightness (L*), redness (a*), and yellowness (b*) parameters. These effects were attributed to the presence of compounds such as chlorophylls, carotenoids, anthocyanins, and other phenolic compounds found in black sapote pulp [38,39]. The stability of these pigments depends on their molecular structure and their interactions with the surrounding hydrocolloid matrix, which can be modified by pH and thermal processing, ultimately influencing color retention during storage [40]. Furthermore, color stability was influenced by pH and processing temperature, resulting in variations toward brownish-green tones at higher pigment concentrations [13].

Overall, these findings suggest that black sapote pigment can be used as a natural alternative to synthetic colorants while also providing nutritionally relevant bioactive compounds.

Fruit vinegar has been recognized as a natural ingredient with potential health benefits, including the reduction in oxidative stress, liver protection, and the regulation of lipid metabolism [41,42,43]. In this context, Székelyhidi et al. [14] developed two gummy formulations based on apple concentrate, incorporating either apple or apricot vinegar with sunflower oil and flavoring compounds. The addition of apple concentrate and vinegar increased the content of organic acids and other bioactive compounds in the gummies.

However, no significant increases in total polyphenol content or antioxidant capacity were observed, which the authors attributed to the thermal degradation of these compounds during processing. Furthermore, the incorporation of vinegar reduced the pH of the formulations, promoting the gelation of the pectin naturally present in the apple concentrate and thereby improving the texture of the product.

The reduction in pH promotes the protonation of pectin carboxyl groups, decreasing electrostatic repulsion between polymer chains, and facilitating junction zone formation. These interactions promote the development of a stronger gel network in the presence of soluble sugars, thereby improving the texture and structural stability of the gummies [35].

The formulations exhibited a total sugar content ranging from approximately 36% to 39%, with fructose as the predominant sugar, followed by glucose and sucrose, consistent with the sugar profile reported for the formulated gummies [14].

Collectively, the available evidence demonstrates that fruits and fruit-derived ingredients represent one of the most versatile approaches for gummy reformulation due to their high content of vitamins, phenolic compounds, anthocyanins, organic acids, soluble sugars, and pectin, which simultaneously enhance nutritional value, antioxidant capacity, and sensory quality [28,29,30,31,32,33,34]. Across studies, a consistent trend was observed in which fruit juices, purées, concentrates, pigments, and fruit-derived products improved color, flavor, antioxidant activity, and physicochemical properties while contributing to gel formation, water retention, texture, and structural stability through interactions between pectin, soluble sugars, organic acids, and the hydrocolloid network [7,11,12,13,14,34,35].

However, common technological challenges include the thermal degradation of phenolic compounds and natural pigments during processing, as well as the need to balance bioactive compound retention with desirable sensory attributes, since formulations with higher functional value were not always the most preferred by consumers [12,13,14]. Differences in fruit composition, phytochemical profile, and processing conditions also contributed to variability in color stability, antioxidant capacity, texture, and overall acceptability. Collectively, these findings highlight the considerable potential of fruits as multifunctional ingredients for developing healthier gummy products, provided that formulation and processing conditions are carefully optimized to preserve both technological performance and consumer acceptance.

## 4. Vegetables

Vegetables constitute a significant source of bioactive compounds beneficial to human health, including dietary fiber, antioxidants, minerals, vitamins, ω-3 fatty acids, and phenolic compounds [44]. In this context, they represent an important source of phytochemicals with antioxidant, anti-inflammatory, and phytoestrogenic properties, making them relevant components of the human diet [45].

Furthermore, their nutritional and biological potential, characterized by the absence of cholesterol and a high micronutrient content, supports their application in the development of value-added food products, such as gummy candies [46].

A representative example is chayote (*Sechium edule*), recognized for its nutritional and functional properties, including antioxidant and phenolic compounds [47]. In addition, it is rich in vitamins, fiber, and potassium, which confer nutraceutical effects [48]. Delgado-Durán et al. [8] developed four gummy formulations enriched with chayote seed, pulp, and peel flour. The results of sensory evaluation showed that two formulations were the most accepted by consumers, corresponding to intermediate concentrations of approximately 6% (F-101) and 9% (F-102) chayote, standing out in attributes such as flavor, sweetness, color, and aroma.

Proximate analysis showed that these formulations exhibited higher contents of dry matter, dietary fiber, protein, and minerals, as well as an increase in reducing sugars (approximately 3%), which can be attributed to the presence of fiber, reducing sugars, water, ash, and proteins in chayote. The increased dietary fiber content may also have contributed to greater water retention and improved structural stability of the gummy matrix through interactions with the hydrocolloid network, thereby influencing texture and reducing moisture migration during storage [49].

Furthermore, thermostability evaluation indicated that a gelatin concentration of approximately 10% was adequate, possibly due to its deformation resistance [8].

The samples remained stable during 6 months of storage, with no evidence of microbial growth or changes in their physical characteristics. Overall, chayote-enriched gummies represent a promising alternative for providing nutritional benefits and offering healthier dietary options [8].

Several studies have explored the use of other vegetables with high nutritional value in the development of functional gummies. Celery (*Apium graveolens*) stands out due to its vitamin, mineral, trace element, and amino acid content, which confers a high nutritional value [50,51].

Ghodsi et al. [15] developed 30 gummy formulations containing celery puree, *Boswellia* spp. (*Boswellia thurifera*) pure gum, and lemon essential oil, which were optimized using response surface methodology. The optimal formulation contained 25% celery puree, 20% *Boswellia* spp. gum, 0.45% lemon essential oil, and approximately 14% sugar.

The incorporation of Boswellia gum significantly increased hardness, chewiness, and gumminess. These improvements can be attributed primarily to the hydrocolloid properties of the gum, whose polysaccharide-rich composition enhances water binding and contributes to the formation and strengthening of the gummy gel network. This behavior is closely related to the high molecular weight and branched structure of *Boswellia* spp. polysaccharides, which promote intermolecular interactions and increase the integrity of the gel network, resulting in enhanced mechanical strength and textural stability [52].

In addition, the inclusion of celery puree and lemon essential oil may have contributed to the overall physicochemical characteristics of the formulation. Furthermore, the optimized gummies exhibited an approximately 85% increase in antioxidant capacity, which was associated with the presence of bioactive compounds, including phenolic compounds, flavonoids, and boswellic acids [15].

Conversely, higher concentrations of sugar (20%) and lemon essential oil (0.5%) increased the adhesiveness and elasticity, likely due to sugar crystallization and the formation of a glossy surface layer. Furthermore, the gummies exhibited a low caloric content and good sensory acceptability [15].

According to the results, vegetables represent a promising source of functional ingredients for gummy reformulation due to their high content of dietary fiber, vitamins, minerals, and bioactive compounds, particularly phenolics and antioxidants [44,45,46,47,48]. Across studies, vegetable incorporation improved nutritional value and enhanced functional properties, mainly through the presence of fiber and polysaccharides that interact with hydrocolloid matrices and improve water retention, gel stability, and texture [8,49].

However, common technological challenges include formulation-dependent changes in hardness, elasticity, and sweetness perception, as well as the need to optimize hydrocolloid concentration and ingredient ratios to maintain desirable sensory and structural properties [15]. Although both chayote- and celery-based formulations demonstrated improvements in antioxidant activity and nutritional composition, differences in ingredient composition and processing conditions led to variability in textural outcomes and consumer acceptability. Overall, vegetable-enriched gummies show strong potential as functional, low-calorie alternatives, provided that formulation parameters are carefully optimized to balance nutritional, technological, and sensory attributes.

## 5. Microalgae

Microalgae represent an important source of bioactive compounds due to their pigment, lipid, protein, amino acid, and other essential nutrient content [53,54]. In particular, their proteins exhibit functional properties such as the ability to form foams, gels, and emulsions, which facilitates their application in various food systems, including 3D-printed foods, baked products, snacks, and dairy alternatives [55]. These functional characteristics are largely attributed to the molecular structure of microalgal proteins and polysaccharides, which enable their interaction with hydrocolloids and contribute to the stabilization of food gels and emulsions [56].

Furthermore, their natural pigment content enables their use as colorants in confectionery products [57]. Interest in their application for developing functional foods, such as gummy candies, has shown a steady increase [58].

An emerging strategy for incorporating microalgae into this type of matrix involves using encapsulated biomass from species such as *Porphyridium cruentum*, *Dunaliella salina*, and *Chlorella vulgaris*. Konar et al. [16] developed 15 gummy formulations enriched with microalgae, observing that their physicochemical and textural properties were influenced by increasing biomass content, which was attributed to their composition rich in carotenoids and chlorophyll. Additionally, improvements in cohesiveness and chewiness were observed, which positively contributed to consumer perception. These textural modifications may also be associated with interactions between microalgal biopolymers, particularly proteins, and the gummies’ polysaccharide matrix. Such interactions can influence gel network formation and stability, thereby affecting textural attributes such as cohesiveness and chewiness [56].

Regarding sensory evaluation, formulations containing 0.40 and 0.50 g/100 g of microalgae obtained the highest scores in color and appearance; however, they exhibited lower flavor acceptability, possibly associated with the presence of phenolic compounds, such as tannins, responsible for astringent or bitter notes. A decrease in the lightness parameter (L*) and an increase in the a* value were observed in terms of color, indicating a light green hue attributed to the presence of microalgae [16].

Overall, gummies enriched with encapsulated microalgal biomass show high potential for the development of products with enhanced functional and sensory properties.

In addition, *Spirulina* sp. (*Arthrospira platensis*) is an important source of bioactive peptides due to its high nutritional value and elevated protein content [53,54]. In this context, Rivera et al. [9] evaluated three gummy formulations prepared using *Spirulina* sp. biomass, sucrose, and freeze-dried açaí pulp (FDAP), with food-grade bovine gelatin (240 Bloom) used as the gelling agent. The formulation containing 1% açaí and 3% *Spirulina* sp. exhibited the best overall performance because of its higher protein, lipid, and mineral contents.

Furthermore, improvements in chewiness and elasticity were observed. These effects may be associated with noncovalent interactions among Spirulina sp. proteins, polysaccharides, polyphenolic compounds, and the gelatin gel network. Such interactions, including hydrogen bonding and hydrophobic interactions, may reinforce the gel structure, thereby improving the textural properties of the gummies.

During sensory evaluation, all three formulations achieved satisfactory acceptability indices ranging from approximately 78–83%. Although the flavor was described as moderate, it remained acceptable, likely because the acidity of the açaí helped mask the characteristic taste of *Spirulina* sp. [9].

In addition, the gummies exhibited a dark coloration resulting from the combined presence of anthocyanins from açaí and the phycocyanin and chlorophyll pigments naturally present in *Spirulina sp*. Finally, stability studies are needed to determine shelf life and optimize storage conditions, including humidity, temperature, and light exposure [9].

Overall, these findings demonstrate the potential of microalgae as functional ingredients for developing nutritionally enriched gummies. Their technological performance appears to be closely associated with the molecular interactions among proteins, polysaccharides, pigments, and the gelatin network, highlighting the importance of structure–function relationships in determining these products’ physicochemical, textural, and sensory properties [59].

Across the available studies, microalgae are promising functional ingredients for gummy reformulation due to their high protein, pigment, lipid, and bioactive compound contents [53,54,55]. Across studies, a consistent trend was observed in which microalgae incorporation improved the nutritional and functional profile of gummies, particularly through increased protein content, enhanced antioxidant potential, and natural color development associated with chlorophylls, carotenoids, and phycocyanin [9,16]. These effects are strongly influenced by the molecular interactions between microalgal proteins, polysaccharides, and the gelatin hydrocolloid network, which contribute to the formation of the gel structure and texture modifications, especially cohesiveness and chewiness [56]. However, common technological challenges include reduced flavor acceptability due to the presence of bitter or astringent compounds and the need to optimize encapsulation and formulation strategies to improve sensory performance [9,16]. Some variability was observed in terms of optimal incorporation levels and sensory outcomes, depending on the microalgal species and co-ingredients used. Overall, microalgae-enriched gummies demonstrate strong potential as functional products, where structure–function interactions play a key role in determining their physicochemical, textural, and sensory properties [59].

## 6. Alternative Sweetening Systems and Fruit Juices

Sweeteners are a key ingredient in gummy formulations because they significantly contribute to their sensory and technological properties [60]. These compounds not only provide sweetness but also contribute to gel structure formation by modulating water availability and promoting interactions within the hydrocolloid network, thereby influencing texture, water activity (a_w_), and product stability [61]. However, synthetic sweeteners may not be suitable for certain consumer groups, such as individuals with diabetes or other conditions that require the restriction or elimination of added sugar intake [27].

This situation has driven the exploration of natural alternatives, such as plant-based sweeteners, including fruit juices, which, although they have lower sweetening power than artificial sweeteners, are considered safer options for consumption [62].

An emerging alternative in this context is the incorporation of fruit juices due to their natural sugar content, such as fructose and sucrose, which can contribute to the sweetness of gummies. Fruits such as sour cherry, pomegranate, and grape have been used for this purpose. In this regard, Pekdogan et al. [17] developed 14 formulations aimed at completely replacing glucose syrup in reformulated gummies.

Fruit juice concentration directly influenced the textural profile, reducing hardness and gumminess, resulting in softer products. This effect is attributed to the higher water content provided by the juice and the presence of natural pectin in the fruits [17]. The presence of naturally occurring pectin, soluble sugars, and organic acids may modify the hydrocolloid network by influencing water binding and gel formation. These interactions contribute to the softer texture of fruit juice-based gummies and illustrate the relationship between the structural composition of fruit ingredients and their technological functionality [35].

In addition, a decrease in pH was observed, which can be explained by the high content of organic acids such as citric, malic, and tartaric acids. Similarly, a_w_ varied due to the interaction between free water and the sugars present in the juice, whereas the density was influenced by the content of soluble solids, including sugars, acids, and polyphenols, which modify the total solids content and the physicochemical characteristics of the gel matrix. The gummies exhibited values of lightness (L*), red–green component (a*), and yellow–blue component (b*), which are associated with the presence of anthocyanins, pigments responsible for the red, purple, and blue hues in fruits [17].

Overall, these results demonstrate that gummies produced with fruit juices have high potential for the development of products with differentiated physicochemical properties and attractive coloration.

Similarly, agave syrup has been used as a natural sweetener in gummy reformulation. In the study by Čižauskaitė et al. [18], four formulations were developed in which this syrup served as the main sweetening agent, complemented with açaí berry extract, grapefruit seed extract, malic acid, sorbitol, vitamin D_3_, and calcium carbonate. The results indicated that the optimal formulation contained approximately 4 g of gelatin and 6 g of agave syrup, determined using response surface methodology.

Agave syrup increased the flexibility and softness of gummies by 27%, an effect associated with its liquid nature and viscous behavior as a natural sweetener. This behavior is associated with the high fructose content and hygroscopic nature of agave syrup, which increases water mobility within the gel matrix and reduces network rigidity, thereby promoting softer and more flexible gummies. In contrast, açaí extract, incorporated to modify color and flavor, did not improve sensory properties due to its polyphenol content (anthocyanins and tannins), which are responsible for astringent and bitter sensations [18].

The addition of calcium and cholecalciferol reduced gummy firmness, which was attributed to calcium interactions within the gelling network and the physical interference of cholecalciferol in the gel structure. In addition, calcium reduced the perceived odor and flavor by reinforcing the gel matrix without promoting the release of aromatic compounds [18].

Overall, the study concludes that gummy-type formulations can be enriched with various functional ingredients; however, flavor-masking strategy optimization must improve sensory acceptability.

Current evidence indicates that alternative sweetening systems, particularly fruit juices and natural syrups, play a key role in gummy reformulation by simultaneously contributing to sweetness, texture, and physicochemical stability [27,60,61,62]. Across studies, a consistent trend was observed in which fruit juices and agave syrup modified the hydrocolloid network by altering water availability, sugar content, and organic acid composition, resulting in softer textures, reduced hardness, and changes in water activity and pH [17,18]. These effects are closely related to the presence of soluble sugars, organic acids, and naturally occurring pectin, which interact with gel-forming agents and influence network structure and stability [35].

However, common technological challenges include variability in sensory acceptance, particularly due to the presence of polyphenolic compounds that may impart bitterness or astringency, as well as the need to balance sweetness intensity with desirable textural properties [18]. Variability among formulations was also observed depending on the type of fruit juice or sweetener used and their compositional differences. Overall, alternative sweetening systems represent promising natural substitutes for refined sugars, although careful optimization is required to achieve an appropriate balance between technological functionality and consumer acceptability.

## 7. Gelling Agents

Gelling agents (starch, pectin, or gelatin) constitute the structural basis of gummies, as they determine their texture, stability, and rheological behavior [10,62]. Their technological functionality depends on their molecular structure and gel-forming mechanisms, which govern water binding, network formation, and the mechanical properties of the final product [59]. Among these, gelatin is one of the most widely used hydrocolloids [63] because of its emulsifying, gelling, and thickening properties [64].

At the industrial level, it is primarily obtained from pork and bovine skins, bones, and demineralized tissues [65]. However, in recent years, there has been a growing interest in the development of plant-based gelling agents, driven by the demand for more sustainable products aligned with plant-based dietary preferences [66].

In this context, cushuro (*Nostoc commune* Vauch) has emerged as a promising gelling alternative. García et al. [19] evaluated three levels of partial substitution of gelatin with freeze-dried cushuro flour (35%, 45%, and 55%) complemented with blueberry pulp. The results showed that the incorporation of 55% cushuro increased protein (approximately 4%) and carbohydrate content (91.58%), while also enhancing the antioxidant capacity of the gummies; meanwhile, fat content decreased to approximately 4%.

Furthermore, the combination of cushuro flour (55%) and blueberry pulp (approximately 13%) increased antioxidant activity by up to approximately 73%, which was attributed to the presence of phenolic compounds, mainly anthocyanins, from both ingredients. Similarly, increasing the proportion of cushuro led to modifications in textural properties, likely due to its polysaccharide and dietary fiber content [19]. These components may modify the hydrocolloid network by enhancing water retention and intermolecular interactions, thereby influencing the textural properties of the gummies and highlighting the relationship between the structural composition of cushuro and its functionality as a gelling ingredient [34].

Variations were observed in the parameters L* (lightness), a* (green–red), and b* (blue–yellow), as well as in C* (chroma) and ΔE (total color difference), attributable to the interaction between natural pigments such as chlorophylls and phycocyanins from cushuro and anthocyanins from blueberry [19].

Overall, the use of underexplored raw materials may represent a promising alternative for improving the functional and technological characteristics of traditional gummies.

Similarly, chicory inulin has been evaluated as a plant-based hydrocolloid in gummy reformulation. Delgado et al. [20] used it as a corn starch substitute in two formulations. The results indicated that the incorporation of inulin produced gummies with a soft, elastic, and slightly sticky texture, associated with its ability to form microcrystals that interact with gelatin. These interactions contribute to the formation of a more homogeneous gel network, thereby improving water retention and providing the soft and elastic texture characteristic of inulin-containing gummies [59].

In addition, higher °Brix values were observed, attributed to the presence of fructans (water-soluble fructose polymers), along with a slight decrease in pH due to mildly acidic compounds. The moisture content remained similar between the formulations, likely due to the high water-holding capacity of inulin [67].

The gummies exhibited a strawberry flavor with sweet and slightly sour notes, as well as a red coloration associated with the use of colorants and flavorings [20]. Chicory inulin is described as a neutral and stable functional ingredient with the potential to act as a co-gelling agent in the development of gummies with reduced caloric content and dietary fiber enrichment. This is particularly relevant for consumers following hypocaloric diets or those requiring control over sugar intake.

Similarly, agar-agar and guar gum have been used as alternatives to gelatin in gummy formulations. Rawat et al. [21] developed a formulation based on both hydrocolloids and turmeric and black pepper. The optimized formulation included 40% sugar, 2% citric acid, 2% turmeric, and 0.6% black pepper. The incorporation of these bioactive compounds resulted in gummies containing approximately 57 mg/100 g of total phenolics and an antioxidant capacity of 37.27%.

Regarding textural properties, increasing the guar gum content (7%) reduced chewiness and gumminess, whereas the combination of agar-agar and guar gum provided a balanced texture in terms of elasticity, cohesiveness, and firmness. This behavior is attributed to the presence of structural polysaccharides (agarose and galactomannans). The complementary functionality of these polysaccharides reflects how differences in molecular architecture influence gel strength, elasticity, and cohesiveness through the formation of interconnected hydrocolloid networks [34].

Texture, appearance, flavor, and overall acceptability were positively evaluated by a semi-trained sensory panel [68].

Overall, this base formulation represents a viable alternative for the development of innovative, functional vegan gummies.

Collectively, these findings support that gelling agents are the core structural components of gummy systems, as their molecular architecture and gel-forming mechanisms determine texture, stability, and rheological behavior [59,62]. A consistent trend was observed across studies in which both animal- and plant-based hydrocolloids modulate the gummy network through water binding, intermolecular interactions, and the formation of three-dimensional gel structures, thereby directly influencing hardness, elasticity, and cohesiveness [19,20,21]. These effects are strongly dependent on the structural characteristics of each hydrocolloid, including polysaccharide branching, molecular weight, and ability to interact with sugars, acids, and bioactive compounds, highlighting the importance of structure–function relationships in gel performance.

However, common technological challenges include achieving textural properties comparable to gelatin when using plant-based alternatives and balancing functional enrichment with desirable sensory attributes such as softness and mouthfeel [20,21]. Some variability among studies was observed depending on the type and concentration of hydrocolloid used, as well as interactions with co-ingredients such as fruit pulps and bioactive extracts. Overall, plant-based and mixed hydrocolloid systems represent promising alternatives for developing vegan and functional gummies, provided that their molecular interactions are carefully optimized to ensure adequate structural and sensory quality.

## 8. By-Products

In recent years, growing concerns about food sustainability have driven interest in the valorization of agro-industrial waste and by-products [11]. In addition to their environmental benefits, many of these by-products contain polysaccharides, dietary fiber, and phenolic compounds that can modify the structure and functionality of GMs, making them promising ingredients for the development of functional confectionery products [34,59]. In this context, one of the most relevant approaches is the utilization of by-products derived from fruits and vegetables, such as gummies, for incorporation into food matrices, thereby contributing to waste reduction and functional food development [66].

An example of this is the use of liquid residues from beetroot (*Beta vulgaris* L.), which have been proposed as a sustainable alternative to glucose syrup. ElObeid et al. [22] subjected these residues to clarification and decolorization processes, achieving a clarity of approximately 77%, which enabled their incorporation into fourteen gummy formulations. The results demonstrated that this by-product is a rich source of bioactive compounds, particularly polyphenols such as phenolic acids and flavonoids.

Furthermore, high values in textural properties were observed, attributable to the sugar and pectin content in the liquid residue of beetroot. A higher content of clarified residue promoted the formation of a firmer gummy structure. This effect is consistent with the ability of pectin and soluble sugars to enhance hydrocolloid interactions and reinforce the gel network, thereby increasing the gummies’ mechanical strength [59].

On the other hand, a shelf-life evaluation of over 8 weeks indicated that the gummies did not exhibit microbiological deterioration [22]. However, the authors emphasize the need for more comprehensive sensory analyses for a thorough product evaluation. Finally, the clarity values obtained for the liquid residue of beetroot demonstrate functionality comparable to that of glucose syrup, positioning it as a sustainable alternative to conventional syrups.

Coffee husk is another by-product of high interest. Boninsegna et al. [23] evaluated the extraction and characterization of bioactive compounds present in coffee silver skin, highlighting its richness in polyphenols, caffeine, dietary fiber, and melanoidins. This extract was incorporated into three gummy formulations at different concentrations (1, 2, and 4 g).

After 120 days of storage, the hardness, chewiness, and stickiness of the samples were affected by the incorporation of the by-product [23], an effect mainly attributed to its fiber, polysaccharides, and phenolic compounds [69], which may modify the gel network, thereby influencing the mechanical properties of the gummies through alterations in hydrocolloid interactions, matrix organization, and water retention [34].

In functional terms, the antioxidant activity of the enriched gummies reached up to 30.25 µmol TE·g^−1,^ and a maximum phenolic compound content was 161.1 mg GAE·kg^−1^. In addition, they exhibited more favorable physical and sensory characteristics than the control group [23].

Overall, these findings suggest that the incorporation of coffee husk into gummies represents a promising alternative to increase their content of bioactive compounds and improve their functional profile.

Yellow pitahaya peels are an abundant and underutilized source of mucilage and betaxanthins, natural pigments with yellow–orange hues and antioxidant properties. Otálora et al. [25] used pitahaya peel mucilage and maltodextrin as encapsulating agents for the microencapsulation of these bioactive compounds, demonstrating that both systems allowed the preservation of a high betaxanthin content and notable antioxidant activity.

Furthermore, FTIR spectroscopy characterization and antioxidant activity evaluation demonstrated that pitahaya peel mucilage contributes to the protection and stability of betaxanthins. Subsequently, the powdered microencapsulates were incorporated into gummy formulations, which were subjected to an in vitro gastrointestinal digestion process, revealing controlled release and stability of the compounds during digestion [25].

In addition, an increase in total dietary fiber content was observed, along with improvements in textural properties, such as gumminess and chewiness, compared with the control sample and gummies made with maltodextrin. These effects may be attributed to the high water-holding capacity of the dietary fiber present in pitahaya peel mucilage, which has been reported to contain soluble fiber and pectic polysaccharides capable of enhancing water retention and reinforcing the gelatin gel network, thereby improving the gummies’ structural integrity and texture [25,26].

These findings illustrate the relationship between the structural characteristics of pitahaya peel mucilage and its functionality as a natural hydrocolloid capable of improving the physicochemical stability of gummy formulations [59]. The gummies also maintained a stable yellow coloration throughout 30 days of storage, confirming the stability of the encapsulation system [25].

Overall, the microencapsulation of betaxanthins using pitahaya peel mucilage represents a promising alternative for the development of functional gummies and the replacement of synthetic colorants in the food industry.

Likewise, green pistachio peel represents another by-product with the potential for gummy reformulation. Roudbari et al. [27] developed three formulations based on green pistachio peel extract, starch, and stevia powder to improve the nutritional value of the product. The formulation was optimized using response surface methodology, determining the use of 2% green pistachio peel extract and 0.026% stevia under optimal conditions.

The results showed that the gummies exhibited high antioxidant activity (IC_50_ = 277 μg·mL^−1^), a total phenolic content of up to approximately 680 mg GAE·100 g^−1^, and a low sugar content (12%). In this regard, the authors highlight that these formulations represent a natural and functional alternative, attributed to the richness in polyphenolic compounds of the pistachio extract [27].

However, further in vivo studies are recommended to validate their biological effects, as well as the application of encapsulation techniques before incorporation, to improve the stability of phenolic compounds.

Similarly, pomegranate peel extract has emerged as a promising alternative in gummy reformulation because of its high content of phenolic compounds, particularly punicalagin and ellagic acid [70]. In this study, two formulations were developed: one prepared with non-microencapsulated pomegranate peel extract (PPE-JG) and another with microencapsulated extract (MPPE-JG).

The results of the chemical analysis showed that the MPPE-JG formulation presented slightly higher values of energy content (approximately 175 kcal/100g), proteins (approximately 10 g/100g), ash (0.21 g/100g), and carbohydrates (approximately 57 g/100g) than the PPE-JG formulation. In the sensory evaluation, no significant differences were observed in flavor and appearance between the two samples; however, the formulation with non-microencapsulated extract, which also exhibited a more intense red coloration, had a slightly higher overall acceptability [70].

Subsequently, the samples were subjected to in vitro evaluation, revealing that the MPPE-JG formulation. However, it showed lower bioaccessibility of total phenolic compounds (approximately 546 mg GAE·100 g^−1^), exhibited 55% higher antioxidant activity as measured by the Oxygen Radical Absorbance Capacity (ORAC) method (approximately 5536 μmol TE·100 g^−1^) compared to the non-ME formulation. This behavior is attributed to the presence of additional bioactive compounds in pomegranate peel, such as quercetin and catechin [70].

Overall, the microencapsulation of pomegranate extract contributes to the protection of bioactive compounds and optimizes their interaction within the gel matrix, thereby improving the stability and functional quality of the gummies. The improved stability achieved through microencapsulation demonstrates how structural modifications of bioactive ingredients can enhance their functionality within hydrocolloid-based gummy systems [59].

Agro-industrial by-products represent a highly promising source of functional ingredients for gummy reformulation due to their richness in dietary fiber, polysaccharides, and phenolic compounds [11,34,59]. Across studies, a consistent trend was observed in which by-product incorporation improved the nutritional and functional profile of gummies, particularly by increasing antioxidant activity, total phenolic content, and dietary fiber, while also contributing to texture and gel structure modifications through enhanced hydrocolloid interactions and water retention [22,23,25,27,70]. These effects are strongly dependent on the structural composition of each by-product and its interaction with the gummy matrix, highlighting the importance of structure–function relationships in determining final product properties.

However, common technological challenges include variability in sensory acceptability, potential changes in texture and color, and the need to improve the stability of bioactive compounds, which are often addressed through processes such as clarification or microencapsulation [25,70]. Some variability among studies was also observed depending on the by-product type and its processing method, particularly regarding sensory preference and retention of bioactive compounds. Overall, agro-industrial by-products offer strong potential as sustainable and functional ingredients for gummies, provided that their structural and physicochemical properties are carefully optimized to ensure product stability and consumer acceptability.

## 9. Current Challenges and Prospects of Gummy Reformulation

### 9.1. Production and Sustainability

Despite their high nutritional value and commercial potential, there is a wide diversity of fruits and vegetables that remain underutilized in the food industry [71]. In developing countries, a significant proportion of these products are lost due to supply chain inefficiencies and the rejection of those that do not meet quality standards, even when they are still suitable for consumption [72].

Additionally, large volumes of byproducts, such as peels, pulp, and seeds, are generated during processing, most of which are discarded [73]. Globally, these residues exceed two billion tons annually [74], representing a major environmental and economic challenge within the food supply chain [75].

However, these by-products have attracted increasing interest due to their high content of bioactive compounds, including phytonutrients with antioxidant activity, as well as their potential health benefits [76]. Consequently, their valorization has gained global relevance, promoting their usage and incorporation into the development of innovative food products [77].

In this context, the availability of high functional value fruits, vegetables, and by-products represents a strategic opportunity for the sustainable development of nutritious and functional gummies. However, the incorporation of these unconventional ingredients poses several scientific, technological, and industrial challenges that have only been partially addressed.

The stability of bioactive compounds during thermal processing and storage is a major challenge that may compromise their functionality [78]. The inclusion of these ingredients can significantly affect the rheological and textural properties of gummies, thereby influencing their sensory acceptability [79].

The intrinsic variability of raw materials, especially in the case of agroindustrial by-products, complicates the standardization of formulations. Limitations related to industrial scalability and increased production costs must also be considered, as they directly impact commercial viability.

Similarly, it is necessary to address aspects related to product shelf life, compliance with regulatory frameworks, and scientific validation of health claims. For instance, regulations established by the United States Food and Drug Administration (FDA), the European Food Safety Authority (EFSA) [80,81], and Regulation (EC) No 2073/2005 [82] must be considered.

In this regard, future research should focus on developing technological strategies to improve the stability and bioavailability of bioactive compounds, optimize their sensory properties, and ensure their reproducibility at an industrial scale. Moreover, a greater integration of multidisciplinary approaches encompassing technological, nutritional, economic, and regulatory aspects is required to consolidate the development of sustainable and competitive functional gummies in the market.

### 9.2. Competition and Innovation

In recent years, the reformulation of gummy confections has experienced significant advances, driven by the growing interest in the use of non-conventional ingredients and the increasing demand for healthier and functional foods, particularly those rich in antioxidant compounds [83]. In this context, various formulations incorporating fruit extracts [7], vegetable extracts [15], microalgae [9], and agro-industrial coproducts [27] have been developed, whose integration promotes the efficient use of resources and improves the nutritional and functional profile of the final product [84].

However, from a competitive perspective, achieving an appropriate balance between functionality and sensory acceptability is one of the main challenges. The incorporation of non-conventional ingredients can significantly modify key attributes of gummy confections, such as texture, taste, aroma, and appearance, directly affecting consumer preference. In this regard, the optimization of formulations and processing conditions is essential to ensure product quality without compromising sensory attributes [6].

Innovation in this type of product is not limited to the incorporation of new ingredients but also involves the development and application of emerging technologies aimed at improving the stability, controlled release, and bioavailability of bioactive compounds [85]. Among these, the integrated liposomal in situ soft sphere (ISSI) technology stands out because it has proven effective in protecting sensitive compounds, such as vitamin C, by enhancing their thermal, structural, and chemical stability during gummy processing and storage [86,87].

Despite these advances, significant challenges remain that limit large-scale industrial adoption, including technological complexity, high costs associated with encapsulation systems, process reproducibility, and scientific validation of functional benefits in consumers. Therefore, future research should focus on developing more efficient, accessible, and scalable technological solutions to strengthen the competitiveness of functional gummy products in a market increasingly oriented toward healthy, sustainable, and innovative foods.

### 9.3. Science and Technology Research

**Figure 2:**
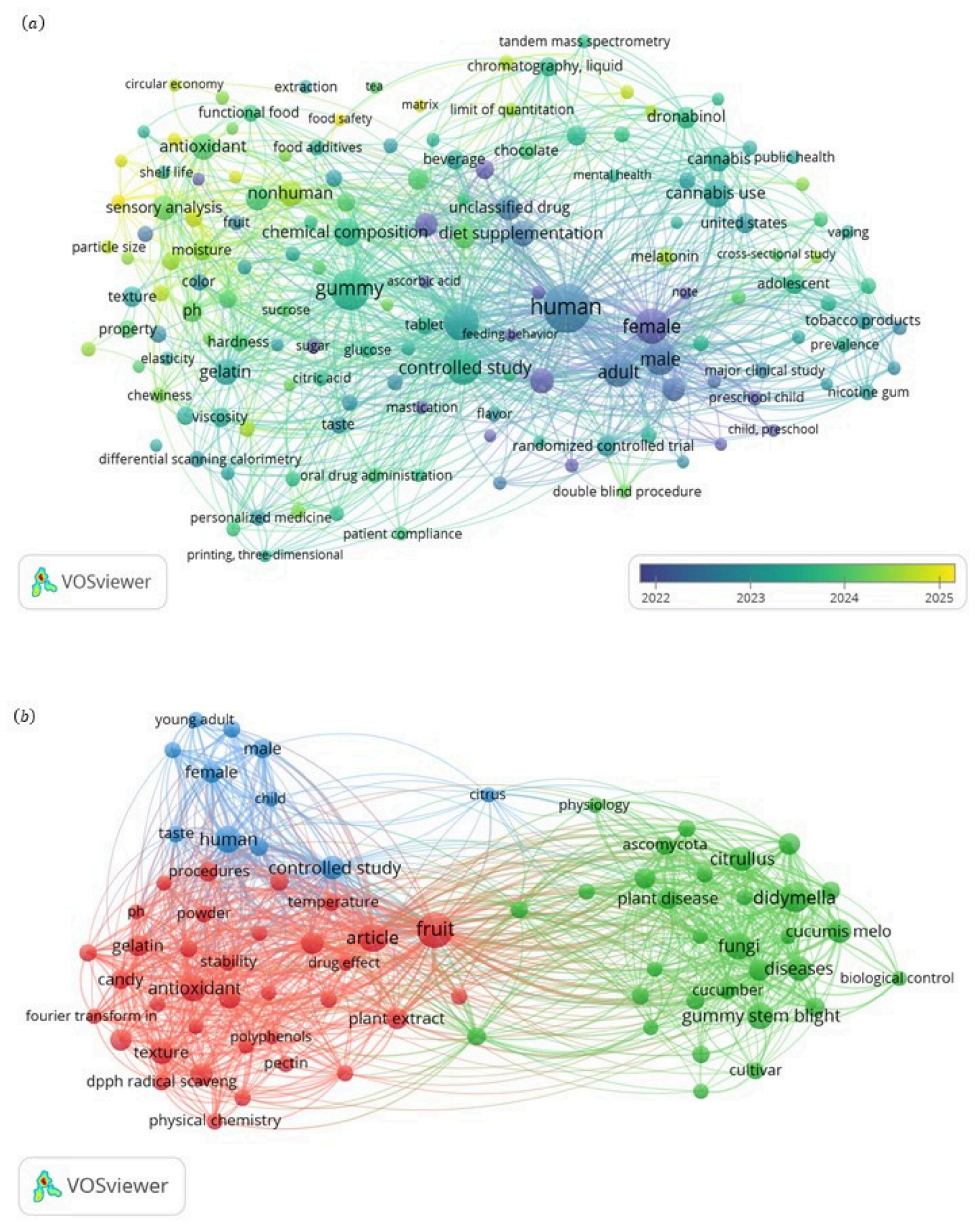

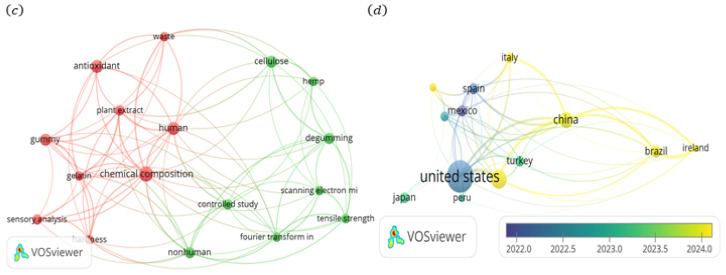
Bibliometric network maps were generated from the Scopus database using VOSviewer (version 1.6.20). (**a**) Keyword co-occurrence network for the general literature on gummy confectionery (“gummy” OR “gummies”; n = 268); (**b**) keyword co-occurrence network for studies on fruit-based gummy confections (“gummies” AND “fruits”; n = 212); (**c**) keyword co-occurrence network for studies on gummies formulated with agro-industrial by-products (“gummies” AND “agro-industrial by-products”; n = 75); and (**d**) country co-authorship network for the general literature on gummy confectionery. Note: The time intervals shown in the figure correspond to the periods with the highest publication output identified by the VOSviewer analysis and do not represent the entire search period. The bibliographic search was conducted on 21 April 2026.

Scientific research on the use of non-conventional ingredients in the reformulation of functional and nutritional gummy confections still shows considerable potential for development. The bibliometric analysis was conducted using the Scopus database. The literature search was performed on 21 April 2026, retrieving publications indexed between 2000 and the search date using the keywords “gummy” OR “gummies” in combination with related terms such as “fruit,” “vegetable,” and “agro-industrial by-products.” Since 2021, a marked increase in publications related to gummies has been observed since 2021, with a total of 268 documents retrieved from Scopus at the time of the search (Figure 2a).

In contrast, studies that specifically focused on the incorporation of non-conventional ingredients, such as fruits (Figure 2b) and agro-industrial coproducts (Figure 2c), remain limited in the Scopus database (2026), highlighting a clear knowledge gap in this field. Most of these studies are recent and mainly concentrated in countries such as China, the United States, Brazil, Ireland, and Italy (Figure 2d).

The bibliometric network analysis based on keyword co-occurrence (Figure 2) reveals the multidimensional structure of the research field. Three main thematic clusters can be identified in the general network (Figure 2a), including food formulation and physicochemical properties (e.g., texture, viscosity, gelatin), bioactive compounds and health-related outcomes (e.g., antioxidants, controlled studies), and clinical or human-centered applications. This distribution reflects the interdisciplinary nature of gummy research, which spans food science and biomedical-oriented studies.

In contrast, the networks focusing on fruits (Figure 2b) and agro-industrial coproducts (Figure 2c) clearly distinguish between formulation-oriented studies and agricultural or biological research domains. This weak integration suggests that raw materials are still primarily investigated outside the gummy confectionery application context.

In particular, agro-industrial coproducts exhibit a low-density and fragmented network, confirming their emerging status within this research area. Finally, the country-level analysis (Figure 2d) indicates a concentration of scientific production in a limited number of countries, mainly China and the United States, suggesting an unequal global distribution of research activity.

## 10. Conclusions

The available evidence demonstrates that the incorporation of unconventional ingredients into gummy reformulation represents a promising strategy to enhance the nutritional and functional quality of these confectionery products. Ingredients such as fruits, vegetables, agro-industrial by-products, algae, and plant extracts have consistently shown strong potential to increase the content of bioactive compounds, including antioxidants, dietary fiber, vitamins, and minerals, while also contributing to improved sensory properties and supporting the development of more sustainable food systems. However, the reported benefits vary depending on the type of ingredient, formulation, and processing conditions, highlighting the need for standardized methodologies that allow more reliable comparisons across studies.

Despite significant advances in the physicochemical, functional, and sensory characterization of reformulated gummies, important knowledge gaps remain. Future research should prioritize assessing the bioaccessibility and bioavailability of bioactive compounds, validating their health benefits through in vivo studies and clinical trials, optimizing processing conditions to minimize compound degradation, and evaluating storage stability under commercial conditions. Furthermore, the development of multifunctional formulations that simultaneously achieve high nutritional quality, adequate technological performance, high sensory acceptability, and extended shelf life is still required. The integration of emerging technologies, such as encapsulation systems, controlled-release strategies, and active and intelligent packaging, also represents a promising research direction to improve product stability and functionality. Furthermore, studies involving agro-industrial by-products should include microbiological analyses to ensure food safety.

Overall, gummy reformulation using unconventional ingredients is a rapidly evolving field with considerable scientific, technological, and industrial potential. Progress in this area will require multidisciplinary collaboration among food scientists, nutritionists, process engineers, and the food industry to develop innovative gummies that combine health benefits, technological functionality, consumer acceptability, and environmental sustainability.

## Figures and Tables

**Table 1:** Effects of unconventional ingredients on the nutritional and functional characteristics of gummy candies.

Non-Conventional Ingredients	Objective	N° Formulations	Formulation Description	Effect on Gummy Characteristics	Statistical Analysis **	Ref
Fruits						
Orange and berries (blackberries, raspberries, strawberries, blackcurrants, and wild blueberries)	Developing gummies using natural ingredients without added sugars or additives	2 + 1 control	Orange juice honey: Orange juice (86.2%), neutral gelatin (3.4%), agar-agar (1.7%) Honey (8.6%). Red berry puree: Red berry puree (54.5%), neutral gelatin (2.7%), agar-agar (1.4%), water (41%), and lemon juice (0.5%).	Orange juice: ↑Energy (up to 73.8 kcal·100 g^−1^), ↑Carbohydrates (up to 4.40 g·100 g^−1^), ↑Protein (up to 4.04 g·100 g^−1^), ↑Fiber (63.95 to 67.07%), ↑Cohesion (89.08 to 91.34%). ↓Moisture (18.2 g·100 g^−1^), ↓Mesophilic Aerobic population (1.0 × 10^2^ CFU·g^−1^), ↓Hardness (3.66 to 4.77 N), ↓Adhesiveness (−0.32 to 0.0 N·s). Red fruit puree: ↑Antioxidant activity (up to 83.7 mg TE·100 g^−1^), elasticity (75.55–81.69%), Gumminess (5.08–5.73 N) and chewability (3.90–4.64 N). ↓Sugar (4.63 g·100 g^−1^), ↓Sodium (0.04 mg·100 g^−1^). Total fat (˂0.5 g·100 g^−1^)	ND	[7]
Pomegranate and apple	To determine consumer perception of pomegranate and apple gummies	12 + 4 controls	Base Gelatin: [13%, 17%, 20%] Variables Pomegranate juice: [27.5%, 29%, 31%, 55%, 58%, 62%] Apple puree: [0%, 27.5%, 29%, 31%] Honey: [25%, 0%] Sucrose: [0%, 25%]	Pomegranate juice + Honey: ↑Chewiness (up to 959 N). Pomegranate juice + Sucrose: ↑DPPH (up to 14.9 mg·100 g^−1^), ↑FRAP (up to 4.59 mg·100 g^−1^), ↑TPC (up to 159 mg·100 g^−1^), ↑ Chewability (up to 900.8 g·100 g^−1^), ↓Adhesiveness (up to −2138 g·s^−1^) Pomegranate juice + apple puree + Honey: ↑Elasticity (up to 1.08%): Pomegranate juice + apple puree + Sucrose: ↑ABTS (up to 3.69 mg·100 g^−1^), ↑Cohesiveness (up to 0.71 g·100 g^−1^), ↑L* (up to 32.7), ↑a* (up to 0.93), ↑b* (up to 5.25) ↓Hardness (7.33 N).	**	[11]
Blueberries and honey	Development of functional gummies based on blueberries and honey	5 + 1 control	Gelatin base: 4 g·100 s g^−1^ Sucrose: [20 g, 40 g, 50 g] and glucose syrup: [0 g, 5 g, 15 g, 25 g] Honey: [0 g, 10 g, 20 g, 30 g] Water: [8 g, 10 g, 16 g, 21 g] Variables Concentrated blueberry juice [0 g (T1, T2, and T3), 30 g (T4), and 60 g (T5)]	Blueberry concentrate juice 60 g: ↑Phenolic content (up to 148.00 mg GAE·100 g^−1^), ↑Flavonoid content (up to 0.51 mg RE·100 g^−1^), ↑Antioxidant activity (IC_50_ = up to >100 mg·mL^−1^). Blueberry concentrate (30 g): ↑Appearance (up to 7.48) ↑Elasticity (up to 6.78%) ↑Firmness (up to 6.56 N), ↑Flavor (Up to 6.86), ↑Gloss (up to 7 34).	**	[12]
Black sapote	Developing gummy bears using pigment from black sapote	4 + 1 control	Base Glucose: 120 g White sugar: 550 g. Sodium benzoate: 1 g. Starch: 3 g. Citric acid: 10 g. Gelatin (290 Bloom): 55 g Water: 200 mL Variables Sapote pigment powder: 1%, 3%, and 5%. Non-fermenting: WF. Fermenting yeast: *L. casei*, *L. rhamnosus*, and *S. cerevisiae.*	1% sapote pigment: ↑L*: 35.88 (WF), ↑a*: 11.68 (WF) 5% sapote pigment: ↑b*: 23.52 (*L. casei*)	**	[13]
Apricot and apple cider vinegar	Development of fruit-based gummies without sugar or sweeteners	2	Apple concentrate juice: Water (30.72%), apple concentrate (46.08%), apple cider vinegar (4.61%), gelatin (15.36%), lemon oil/sunflower oil (2.30%), and apple flavor (0.92%). Apricot concentrate juice: Water (30.72%), apple concentrate (46.08%) (4.61%), gelatin (15.36%), Lemon oil/sunflower oil (2.30%), apricot flavor (0.92%).	Apple concentrate juice: ↑Gumminess (up to 3266.25 N·m^−2^) ↑Chewiness (up to 3425.33 N·m^−2^). Apricot concentrate juice: ↑TAC (up to 0.75 mg AAE·100 g^−1^), ↑TPC (up to 1.46 mg GAE·100 g^−1^), ↑Cohesion (up to 0.834 J·m^−3^), ↑Elasticity (up to 0.951 N·m^−2^) ↓pH (4.36), ↓Hardness (3341.67 N·m^−2^).	**	[14]
	Vegetables					
Chayote	Gummies enriched with high-value ingredients	4	Base Guar: [0.31%, 0.31%, 0.31%, 0.44% m/m] Citric acid: [0.31%, 0.31%, 0.44%, 0.31%] Ascorbic acid: [0.06%, 0.12%, 0.06%, and 0.018%] Agave syrup: [13.5%, 18%, 9%, 13.5%] Gelatin: [10.4%, 10.4%, 11.3%, 9.4%] Food coloring: 3 drops Variable Chayote flour: [6.3% (F-101), 8.8% (F-102), 6.3% (F-103), 7.5% (F-104)].	F-101 (6.3%): ↑Fiber (up to 9.62%), ↑Protein (up to 1.70) F-102 (8.8%): ↑Solids (up to 23.71%), ↑Reducing sugars (up to 2.97%), ↑Ash (up to 3.14%). ↓Water (74.42%).	ND	[8]
Celery, *Boswellia* spp. gum, and lemon essential oil	Produce function of vegan gummies	30	Base: Emulsifier lecithin E47: 0.1% Essential lemon: [0.25%, 0.375%, 0.5%]. Sugar: [10%, 15%, 20%]. *Boswellia* spp.: [10%, 15%, 20%]. Variable: Celery puree: [25%, 37.5%, 50%]. *Boswellia* spp.: [10%, 15%, 20%].	Celery puree 25%: ↓pH (4.89), ↓Hardness (25.4 N), ↓Adhesiveness (0.95 N). Celery puree 37.5%: ↑Sugar (up to 29.84 g·100 g^−1^), ↑Elasticity (−0.95 N), ↑Overall acceptance (4.86). Celery puree 50%: ↑Energy (up to 127.58 kcal·100 g^−1^), ↑Chewability (63.13 N), ↑Chewiness (up to 75.86 N), ↑Antioxidant capacity (85.58%). ↓ a_w_ (0.692). *Boswellia* spp. gum 10%: ↑Elasticity (up to 0.91 N). ↓Hardness (25.40 N), ↓Adhesiveness (−0.91 N). *Boswellia* spp. gum 15%: ↑Chewability (up to 66.23 N), ↑Chewiness (75.86 N), ↑Overall acceptance (up to 4.67). *Boswellia* spp. gum 20%: ↑Energy (up to 127.58 kcal·100 g^−1^), ↑Sugar (up to 25.3 g·100 g^−1^), ↑Antioxidant capacity (85.58%).	**	[15]
	Microalgae and marine sources					
*Porphyridium cruentum*, *Dunaliella salina*, and *Chlorella vulgaris*	Evaluate the potential application of three species of encapsulated microalgae	15 + 3 controls	Base Water: 17.4 g·100 g^−1^ Sucrose: 32.6 g·100 g^−1^. Glucose syrup: 41.7 g·100 g^−1^ (42 DE). Solution of gelatin (250 Bloom, 1:2): 6.50 g·100 g^−1^. Citric acid (1:1): 1.80 g·100 g^−1^. Variable Encapsulated microalgae: [0.10, 0.20, 0.30, 0.40 y 0.50 g·100 g^−1^].	Encapsulated microalgae 10%: ↑L* (up to 39.23), ↑Chromaticity matrix: (up to 91.24). ↓Hardness (20.35 N). Encapsulated microalgae 20%: ↑Elasticity (up to 1.44%), ↑Resilience (up to 0.43%), ↑Chewability (up to 306.4 N), ↑b* (up to 16.46), ↑Chroma (up to 16.48). Encapsulated microalgae 30%: ↑Carotenoids (up to 1.83 µg·g^−1^), ↑Chlorophylls (up to 0.89 µg·g^−1^), ↑Cohesion (up to 0.97%). Encapsulated microalgae 40%: ↓ a_w_ (0.663). Encapsulated microalgae 50%: ↑a* (up to 5.73), ↑Viscosity (up to 3041.2 g·100 g^−1^) ↓Moisture (10 g·100 g^−1^).	**	[16]
*Spirulina sp.* and *açaí*	Develop gummies without sucrose or artificial colors	3 + 1 control	*Spirulina* sp. biomass 1% and *açaí* 3%: Gelatin (10 g·100 g^−1^), water (27 g·100 g^−1^), *Spirulina* sp. (1 g·100 g^−1^), *açaí* pulp (3 g·100 g^−1^), maltitol (33 g·100 g^−1^), erythritol (12 g·100 g^−1^), isomalt (12 g·100 g^−1^), citric acid (1 g·100 g^−1^), acai flavor (1 g·100 g^−1^). *Spirulina* sp. biomass 3% and *açaí* 1%: Gelatin (10 g·100 g^−1^), water (27 g·100 g^−1^), *Spirulina* sp. (3 g·100 g^−1^), *açaí* pulp (1 g·100 g^−1^), maltitol (33 g·100 g^−1^), erythritol (12 g·100 g^−1^), isomalt (12 g·100 g^−1^), citric acid (1 g·100 g^−1^), açaí flavor (1 g·100 g^−1^). *Spirulina* sp. biomass 2% and *açaí* 2%: Gelatin (10 g·100 g^−1^), water (27 g·100 g^−1^), *Spirulina* sp. (2 g·100 g^−1^), *açaí* pulp (2 g·100 g^−1^), maltitol (33 g·100 g^−1^), erythritol (12 g·100 g^−1^), isomalt (12 g·100 g^−1^), citric acid (1 g·100 g^−1^), açaí flavor (1 g·100 g^−1^).	*Spirulina* sp. biomass 1% and *açaí* 3%: ↑Carbohydrates (up to 84.99 g·100 g^−1^), ↑Phenolic content (up to 0.254 mg GAE g^−1^), ↑Gum content (up to 17.03 N), ↑Cohesion (up to 0.96), ↑L* (up to 19.23), ↑a* (up to 7.53). ↓Ash (0.82 g·100 g^−1^). *Spirulina sp.* biomass 3% and *açaí* 1%: ↑Protein (up to 13.11 g·100 g^−1^), ↑Chewability (up to 16.61 N), ↑Resilience (up to 0.82), ↑Elasticity (up to 0.99 mm). ↓Lipids (1.18 g·100 g^−1^), ↓Adhesiveness (−0.43 N). *Spirulina* sp. biomass 2% and *açaí* 2%: ↑Cohesion (up to 0.96%), ↑DPPH (up to 52.9%), ↑ABTS (up to 33.7%), ↑b* (up to 11.23). ↓Hardness (16.49 N).	**	[9]
	Alternative sweetening systems and fruit juices					
Sour cherry, pomegranate, and grape	Evaluate the effects of fruit juice concentrate formulations	14	Base Gelatin: 22.50% Sucrose: 35% Variables Sour cherry juice: [0%, 11.54%, 33.85%, 67.69%] Pomegranate juice: [0%, 11.54%, 33.85%, 67.69%]. Grape juice: [0%, 10.91%, 22.14%, 67.69%].	Sour cherry juice: ↓ a_w_ (0.46) Orange juice: ↑a* (up to 11.2), ↑b* (up to 6.28) ↓pH (2.22) Grape juice: ↑Viscosity (up to 947.94 g·100 g^−1^) Sour cherry juice + Orange juice: ↑ Elasticity (up to 0.99%) ↓Hardness (146.1 g·100 g^−1^). Sour cherry juice + Orange juice + Grape juice: ↑Cohesion (up to 1.19), ↑Density (up to 1.53 g·mL^−1^). ↑L* (up to 30.7).	**	[17]
Agave and *açaí*	Make gummies and evaluate the influence of their ingredients	4 + 1 control	C1: Gelatin (4.3 g), water (24.6 g), agave syrup (6.3 g), sorbitol (4.0 g), grapefruit seed extract (0.2 g), malic acid (0.6 g). C2: Gelatin (4.3 g), water (20.8 g), agave syrup (6.3 g), sorbitol (4.0 g), grapefruit seed extract (0.2 g), malic acid (0.6 g), vitamin D3 (0.8 g), calcium carbonate (3.0 g). C3: Gelatin (4.3 g), water (24.1 g), agave syrup (6.3 g), sorbitol (4.0 g), grapefruit seed extract (0.2 g), malic acid (0.6 g), acai berry extract (0.5 g). C4: Gelatin (4.3 g), water (20.3 g), agave syrup (6.3 g), sorbitol (4.0 g), grapefruit seed extract (0.2 g), malic acid (0.6 g), vitamin D3 (0.8 g), calcium carbonate (3.0 g), acai berry extract (0.5 g).	Agave syrup: Firmness <24.71 to 90.41 g>, Strength <51.64 to 378.11 g>, Hardness <67.30 to 726.88 g> ↑Color: C3 (4.54), Odor: C3 (4.15). ↑Quality: C2 and C4.	**	[18]
	Gelling agents					
Blueberries and cushuro	Improving gummies made with blueberry and cushuro	3 + 1 control	Blueberry pulp 12.74% + Cushuro flour 35%: Sugar (31.77%), Glucose (38.64%), blueberry pulp (12.74%), water (11.16%), citric acid (0.11%), unflavored gelatin (35%), cushuro flour (35%). Blueberry pulp 12.74% + Cushuro flour 45%: Sugar (31.77%), Glucose (38.64%), Blueberry pulp (12.74%), water (11.16%), citric acid (0.11%), unflavored gelatin (45%), cushuro flour (45%). Blueberry pulp 12.74% + Cushuro flour 55%: Sugar (31.77%), glucose (38.64%), blueberry pulp (12.74%), water (11.16%), citric acid (0.11%), unflavored gelatin (55%), cushuro flour (55%).	Blueberry pulp 12.74% + Cushuro flour 35%: ↑L* (up to 33.71), ↑a* (up to 0.85). Blueberry pulp 12.74% + Cushuro flour 45% ↑ L* (up to 33.71). ↓Ash (0.09%). Blueberry pulp 12.74% + Cushuro flour 55%: ↑Protein (up to 3.85%), ↑Carbohydrates (up to 91.58%), ↑Antioxidant capacity (up to 73.46%), ↑b* (up to 0.77). ↓Fat (4.13%)	**	[19]
Chicory inulin	Determine the effect of replacing starch with inulin	2	Starch: Glucose syrup (790 g), water (263 g), sucrose (208 g), starch (90 g), pork skin gelatin A (35 g), citric acid (9 g), lactic acid (5 g), sodium citrate (1 g), vitamins (2 g), attractive red coloring (2 g), and strawberry flavoring (2 g). Chicory inulin: Glucose syrup (790 g), water (263 g), sucrose (208 g), inulin (90 g), pork skin gelatin (35 g), citric acid (9 g), lactic acid (5 g), sodium citrate (1 g), vitamins (2 g), attractive red coloring (2 g), and strawberry flavor (2 g).	Starch: ↑Chewiness (up to 80.6 N·mm^−1^), ↑Gummy texture (up to 19.3 N), ↑ Cohesion (up to 0.7 N) ↓ Moisture (177 to 180 g·kg^−1^) Chicory inulin: ↑Soluble solids (up to 80.7°Brix), ↑Elasticity (up to 4.35 mm), ↑Cohesion (up to 0.7 N), ↑L* (up to 32.0), ↑a* (up to 34.4), ↑b* (up to 4.3), ↑Flavor (up to 3.4). ↓pH (3.22), ↓Hardness (24.5 N).	**	[20]
Agar-agar and guar gum	Evaluate the impact of replacing gelatin with plant- based hydrocolloids on the texture of the gummies	1 + 1 control	Sugar: 40% Citric acid: 2% Turmeric: 2% Black pepper: 0.6% Agar-agar: 1% Guar gum: 5.5%	Agar-agar + guar gum: Moisture (11.21%), a_w_ (0.71), Brix (68.5), pH (3.5), Total phenols (56.9 mg·100 g^−1^), Antioxidant capacity (DPPH) (37.27%), Curcumin (0.054%), Piperine (0.02%), Hardness (39.46 N), Chewability (1332.37 N), Gumminess (2105.013 N), Elasticity (0.62 mm)	**	[21]
	By-products					
Beet processing residue	Developing an alternative to glucose syrup from liquid beet waste	14 + 1 control	Base Glucose syrup: [0 g, 10.20 g, 13.23 g, 14.13 g, 20.13 g, 20.85 g 26.47 g, 27.57 g, 30.05 g, 39.70 g, 41 g]. Gelatin: [5 g, 5.47 g, 5.70 g, 6.45 g, 7 g]. Sucrose: 42.27 g Water: 1:2 Variable Liquid beet residue: [0 g, 0.23 g, 10.20 g, 13.23 g, 20.13 g, 20.85 g, 26.47 g, 27.57 g, 30.05 g, 39.70 g, 41 g].	↑Liquid beet residue: ↑Gumminess (up to 2653.99 N), ↑Chewiness (up to 1732.43 N), ↑b* (up to 11.0), ↑a* (–0.45), ↑TPC (45.65 mg GAE·100 g^−1^) ↓pH (4.28), ↓ Moisture (10.71 g·100 g^−1^). Up to 20.13 g·100 g^−1^: ↑Elasticity (up to 69.37%), ↑Cohesion (up to 97.93%). ↑TSS (up to 88.7°Brix), ↑L* (up to 96.7). ↓Hardness (9.97 N).	**	[22]
Coffee husk	Evaluate the applicability of coffee husks in the formulation of gummies	3 + 1 control	Coffee husk extract 1 g: Sucrose (31 g), glucose syrup (28 g), pork gelatin (8 g), apricot juice (22 g), citric acid (1 g), coffee husk (1 g), and water (9 g). Coffee husk extract 2 g: Sucrose (31 g), glucose syrup (28 g), pork gelatin (8 g), apricot juice (22 g), citric acid (1 g), coffee husk (2 g), and water (8 g). Coffee husk extract 4 g: Sucrose (31 g), glucose syrup (28 g), pork gelatin (8 g), apricot juice (22 g), citric acid (1 g), coffee husk (4 g), and water (6 g).	Coffee husk extract 1 g: ↑Cohesion (up to 0.95), ↑Chewability (up to 74.40 N·mm), ↑L* (up to 48.3), ↑a* (up to 2.8), ↑b* (up to 6.06). ↓Hardness (2.41 N), ↓ a_w_ (0.55), pH (3.89). Coffee husk extract 2 g: ↑Elasticity (up to 0.95 mm), ↑Cohesion (up to 0.95), ↑Stickiness (−1.04 N). Coffee husk extract 4 g: ↑ TSS (up to 65.14°Brix), ↑ TPC (up to 295.10 mg GAE·100 g^−1^), ↑ABTS (up to 41.20 µmol TE·100 g^−1^), ↑Cohesion (up to 0.95), ↑L* (48.3). ↓Moisture (13.63 g·100 g^−1^).	**	[23]
Watermelon exocarp	Determine the physicochemical and sensory properties of the gummies	12 + 1 control	Base Pectin: 0.541% Gum arabic: 0.541% Sucrose: 20% Glucose: 10% Starch: 0.18% Citric acid: [0.75%, 1%]. Agar: [0.5%, 1%]. Water: [16.738%, 16.988%, 17.238%, 17.488%, 31.738%, 31.988%, 32.238%, 32.488%, 46.738%, 46.988%, 46.738%, 46.988%, 47.238%, 47.488%]. Variables Watermelon exocarp powder: [20%, 35%, 50%].	Watermelon rind powder 20%: ↑Elasticity (up to 5.355 mm). ↓Hardness (16.35 N) Watermelon exocarp powder 35%: ↑Cohesion (up to 0.730), ↑L* (up to 83.18). Watermelon exocarp powder 50%: ↑Viscosity (up to 1.55 Pa·s), ↑Chewiness (up to 125.35 g·mm), b* (up to 24.55), ↑Gumminess (up to 29.65 N), ↑a* (−9.97) ↓Moisture (23.348 g·100 g^−1^), ↓ a_w_ (0.695), pH (3.02).	**	[24]
Yellow pitahaya peels	Incorporate powdered microencapsulates into the gummy formulation	2 + 1 control	Microencapsulated pitahaya peel mucilage: Glucose syrup (31%), sucrose solution (34%), gelatin (8%), citric acid (0.3%), microencapsulated pitahaya peel mucilage (2.0%). Microencapsulated maltodextrin: Glucose syrup (31%), sucrose solution (34%), gelatin (8%), citric acid (0.3%), microencapsulated maltodextrin (2.0%).	Microencapsulated pitahaya peel mucilage: ↑Elasticity (up to 0.97), ↑Cohesion (0), ↑Adhesiveness (up to 450.9). ↓Hardness (1.74 N). ↑TDFC (up to 0.82 g·100 g^−1^) Microencapsulated maltodextrin: Gumminess (up to 3.05 N), ↑Chewiness (up to 2.94 N), ↑Elasticity (up to 0.97), ↑ORAC (528.2 µmol Trolox equivalents·g^−1^)	**	[25]
Green pistachio shells, stevia, and starch	Improving the nutritional value of gummies	3 + 1 control	F1: Green pistachio shell (2%), gelatin (9%), starch (1%), Stevia (0.026%). F2: Green pistachio shell (2%), gelatin (2%), starch (8%), Stevia (0.026%). F3: Green pistachio shell (2%), gelatin (3%), starch (7%), Stevia (0.040%).	Green pistachio shell extract + Starch + Stevia: Moisture <13.00% to 17.95%>, pH <3.5–6.5> Hardness <1967 g to 3572 g>, Elasticity <2.0 mm to 3.4 mm>, Cohesion <0.75 to 1.02>. Phenolic content (680.31 mg GAE·100 g^−1^), Antioxidant activity (277 μg·mL^−1^).	**	[26]
Pomegranate peel extract	Characterize a prototype of gummies incorporating PPE or MPPE	2	Non-microencapsulated pomegranate peel extract: Oligofructose syrup, maltitol, water, gelatin, non- microencapsulated pomegranate peel extract, citric acid, and Stevia powder. Microencapsulated pomegranate peel extract: Oligofructose syrup, maltitol, water, gelatin, microencapsulated pomegranate peel extract, citric acid, and Stevia powder.	Non-microencapsulated pomegranate peel extract: ↑Protein (up to 9.67 g·100 g^−1^), ↑Ash (up to 2.27 g·100 g^−1^), ↑TPC (up to 372.7 mg GAE·100 g^−1^), ↑DPPH (up to 434.8 µmol TE·g^−1^), ↑FRAP (up to 1961.1 µmol TE·g^−1^), ↑ORAC (up to 3500.5 µmol TE·g^−1^). ↓Fat (1.18 g·100 g^−1^). Microencapsulated pomegranate peel extract: ↑Energy (up to 174.9 kcal·100 g^−1^), ↑Carbohydrates (56.99 g·100 g^−1^). ↓Moisture (11.02%).	**	[27]

DPPH: 2,2-diphenyl-1-picrylhydrazyl. FRAP: Ferric Reducing Antioxidant Power. TPC: Total phenolic content. ABTS: 2,2′-azino-bis (3-ethylbenzothiazoline-6-sulfonic acid), TAC: Total antioxidants, TSS: Total soluble solids. ORAC: Oxygen Radical Absorbance Capacity. WF: Non-fermenting. TDFC: Total Dietary Fiber Content. PPE: Pomegranate peel extract. MPPE: Microencapsulation of pomegranate peel extract. The symbols ↑ and ↓ indicate directional changes (increases or decreases, respectively) that were reported in the respective original studies. ** *p* < 0.05: Significance level. ND: Not determined.

## Data Availability

No new data were created or analyzed in this study. Data sharing is not applicable to this article.
